# Dysregulated human Tyrosyl-DNA phosphodiesterase I acts as cellular toxin

**DOI:** 10.18632/oncotarget.13528

**Published:** 2016-11-23

**Authors:** Selma M. Cuya, Evan Q. Comeaux, Keith Wanzeck, Karina J. Yoon, Robert C.A.M. van Waardenburg

**Affiliations:** ^1^ Department of Pharmacology and Toxicology, University of Alabama at Birmingham, Birmingham, AL 35294-0019, USA; ^2^ Department of Pathology, St. Jude Children's Research Hospital, Memphis, TN 38105-3678, USA; ^3^ Department of Medicine, Division of Clinical Immunology & Rheumatology, University of Alabama at Birmingham, Birmingham, AL 35294-0001, USA

**Keywords:** TDP1, DNA-adducts, biochemistry, DNA topoisomerases, DNA repair

## Abstract

Tyrosyl-DNA phosphodiesterase I (TDP1) hydrolyzes the drug-stabilized 3’phospho-tyrosyl bond formed between DNA topoisomerase I (TOPO1) and DNA. TDP1-mediated hydrolysis uses a nucleophilic histidine (His^nuc^) and a general acid/base histidine (His^gab^). A Tdp1His^gab^ to Arg mutant identified in patients with the autosomal recessive neurodegenerative disease SCAN1 causes stabilization of the TDP1-DNA intermediate. Based on our previously reported His^gab^-substitutions inducing yeast toxicity (Gajewski et al. J. Mol. Biol. 415, 741-758, 2012), we propose that converting TDP1 into a cellular poison by stabilizing the covalent enzyme-DNA intermediate is a novel therapeutic strategy for cancer treatment. Here, we analyzed the toxic effects of two TDP1 catalytic mutants in HEK293 cells. Expression of human Tdp1His^nuc^Ala and Tdp1His^gab^Asn mutants results in stabilization of the covalent TDP1-DNA intermediate and induces cytotoxicity. Moreover, these mutants display reduced *in vitro* catalytic activity compared to wild type. Co-treatment of Tdp1^mutant^ with topotecan shows more than additive cytotoxicity. Overall, these results support the hypothesis that stabilization of the TDP1-DNA covalent intermediate is a potential anti-cancer therapeutic strategy.

## INTRODUCTION

**Figure 1:**
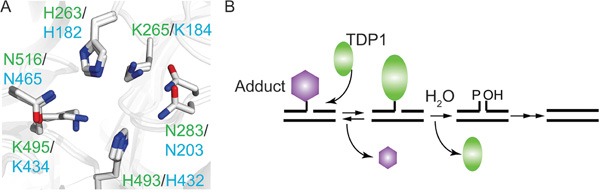
The TDP1 catalytic mechanism is conserved from yeast to human **A**. Overlay of yeast and human crystal protein structure showing the catalytic HxK-motif; labeled in green are human TDP1 residue numbering (PDB # 1NOP [2]) and in cyan the yeast TDP1 residue numbering (PDB# 1Q32 [4]). His263 in hTDP1 and His182 of yTDP1 represent the nucleophilic Histidine (His^nuc^), while His493 of hTDP1 and His432 in yTDP1 represent the general acid/base Histidine (His^gab^). **B**. TDP1 two-step catalytic mechanism. Step 1 (Formation): TDP1 resolves the DNA-adduct via nucleophilic attack of the His^nuc^ that releases the adduct and generates a TDP1-DNA covalent complex via the 3’phospho-amide bond. Step 2 (Dissociation): His^gab^ activates a water molecule that hydrolyze the TDP1-DNA linkage, dissociating TDP1 from the DNA, leaving behind a single-strand nick, which requires further processing by polynucleotide kinase-phosphatase prior to DNA ligation.

Tyrosyl-DNA phosphodiesterase I (TDP1) hydrolyzes 3’phospho-adducts and to a limited extent 5’phospho-adducts within DNA strand breaks (reviewed in [1]). Tdp1 catalysis is structurally and mechanistically conserved from yeast (y) to human (h), and centers on the formation and resolution of a requisite covalent TDP1-DNA reaction intermediate, also called a TDP1-DNA covalent complex (TDP1-cc) (Figure 1). This two-step cycle utilizes spatial and temporal coordinated action of two catalytic histidines [2–8]. During step 1, the nucleophilic histidine (His^nuc^: hTDP1His^263^ and yTDP1His^182^) attacks the DNA 3’phospho-adduct bond to form a TDP1-cc. In step 2, the second histidine functions as a general acid/base (His^gab^: hTDP1His^493^ and yTDP1His^432^) and activates water to hydrolyze the 3’phospho-hystidyl (TDP1His^nuc^-DNA) linkage, dissociating TDP1 from the DNA (Figure 1) [2–8].

Besides the DNA repair enzyme TDP1, DNA topoisomerases (TOPOs; TOPO is used instead of the official abbreviation “TOP” to emphasize the difference between the protein names TDP1 and TOP1) that adjust DNA topology form a transient covalent enzyme-DNA reaction intermediate (TOPO-cc) during their catalytic cycle (review in [9–11]). However, this transient covalent enzyme-DNA intermediate can potentially result in toxic DNA lesions. This paradigm is extensively exploited by cancer-chemotherapeutics that induce toxicity via stabilization of the enzyme-DNA intermediate, e.g. etoposide for TOPO2 and camptothecin (CPT) for TOPO1, including FDA-approved CPT analogs topotecan and irinotecan [12, 13].

Most enzymes that form a covalent-phosphoryl reaction intermediate with a DNA-end are potential substrates for TDP1. Hence, TDP1 is able to hydrolyze 3’phospho-tyrosyl (TOPO1-DNA), 3’phospho-hystidyl (TDP1-DNA), and 5’phospho-tyrosyl (TOPO2-DNA) linkages [3, 4, 11, 13–21]. Moreover, TDP1 is also able to hydrolyze smaller substrates predominantly from the 3’-end of the DNA but also 5’ oriented adducts including damaged nucleotides, such as 3’phospho-glycolates or 5’- and 3’-abasic sites generated by oxidative damage, γ-irradiation and/or chain terminating nucleosides, e.g. cytarabine and acyclovir [4, 18, 22–27]. Thus, TDP1 is a versatile DNA repair enzyme involved in the removal of DNA-adducts generated by a wide variety of chemotherapeutics and endogenously generated toxins.

Stabilization of enzyme-DNA intermediates can also be achieved via the introduction of mutations. For example, there is a naturally occurring Tdp1His^gab^Arg (hTdp1H^493^R) mutation identified in patients with the neurodegenerative disease spinocerebellar ataxia with axonal neuropathy (SCAN1) [28]. This point mutation enhances the stability of the covalent Tdp1^H493R^-cc due to delayed hydrolysis of the 3’phospho-histidyl bond [3, 29]. Moreover, the His^gab^Arg mutant enzyme characteristics are conserved from human to yeast. Some of those characteristics include increased stability of the TDP1-DNA complex that coincides with a reduced catalytic activity *in vitro* and increased cell sensitivity to DNA damaging agents, such as CPT, bleomycin, H_2_O_2_, γ-irradiation, and chain-terminating nucleoside analogs [3, 4, 22, 30, 31]. Additionally, we have identified alternative substitutions of yTDP1His^gab^, including yTdp1H^432^N, that induce more severe cellular toxicity than the yeast SCAN1-mutant analog (yTdp1H^432^R) [3, 4]. Moreover, the yTdp1H^432^N mutant formed stable TDP1-DNA intermediates in yeast [3, 4]. These observations suggest that TDP1-DNA intermediate stabilization by catalytic dysregulation converts yTDP1 into a cellular poison, exposing a potential novel anti-cancer therapeutic strategy.

## RESULTS

### Expression of hTDP1 mutants in yeast induces hTOPO1-dependent toxicity

**Figure 2:**
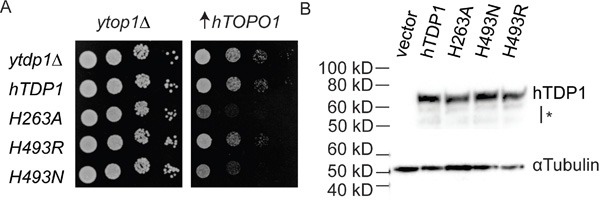
hTDP1 catalytic mutants induce hTOPO1-dependent toxicity **A**. *top1*Δ,*tdp1*Δ cells were co-transformed with vector control (y*top1*Δ) or YCpGAL1-hTOP1·U (↑h*TOPO1*) and control vector (y*tdp1*Δ) or the indicated YCpGAL1-h*TDP1*·L plasmid. Exponentially growing cultures were diluted to an OD_595_ of 0.3 and ten-fold serially diluted and spotted onto selective galactose plates. Plates were incubated at 30°C for 4 days. **B**. Total cell extracts from galactose induced *top1*Δ,*tdp1*Δ cells co-transformed with vector control (y*top1*Δ) and the indicated YCpGAL1-h*TDP1*·L or its control vector (vector) used in (A) were resolved on 12% SDS-PAGE and stained with anti-human TDP1 and with anti-**α**Tubulin. *: partly truncated hTDP1 proteins. Blot was cropped and exposure levels were not edited.

To examine if the molecular basis of the TDP1-induced toxicity of yTDP1 catalytic mutants [3, 4, 32] is a conserved property of the TDP1 family, we generated the analogous human His^nuc^Ala (H^263^A) and His^gab^Arg/Asn (H^493^R/N) substitutions. Mutant hTDP1 alleles were expressed from plasmid-borne galactose-inducible (*GAL1*) promoter constructs. To facilitate species-specific interactions, hTOPO1 was also expressed from the *GAL1*-promoter but borne on a different plasmid. Co-expression of the hTDP1 mutants with hTOPO1 in a *TOP1*, *TDP1* deleted (*top1*Δ,*tdp1*Δ) yeast strain [32] induced a TOPO1-dependent cellular toxicity (Figure 2A). Expression of the hTdp1H^493^R mutant had marginal effects on yeast cell viability, similar to wild type hTDP1, while hTdp1H^493^N and hTdp1H^263^A expression induced toxicity (Figure 2A). The expression level of the catalytic mutants was similar to wild type, suggesting that the phenotypes are due to alterations in catalytic function rather than a difference in expression (Figure 2B) or expression levels of yeast and human TOPO1 (shown in [3]). The observed phenotypes for these hTDP1 mutants are not as robust as the analogous yeast mutants, which is likely due to the expression of human enzymes in yeast.

### TDP1 catalytic mutants reduce colony formation of HEK293 cells

The phenotypes induced by the hTdp1 mutants in our yeast model imply a conserved mechanism underlying the substrate (TOPO1)-dependent toxicity caused by dysregulated TDP1 enzymes [3, 4, 32]. To determine if these TDP1 mutant enzymes induce toxicity in human cells, we stably transfected HEK293T-REx (HEK293Tetracycline-Regulated Expression) cells with control (no h*TDP1*), wild-type h*TDP1*, h*Tdp1H^493^N*, and h*Tdp1H^263^A* expression vectors. The HEK293T-REx cells contain the Tet-on expression system in which doxycycline (Dox) treatment induces ectopic h*TDP1*, h*Tdp1H^493^N*, and h*Tdp1H^263^A* expression from a modified *CMVTetO_2_* promoter. Moreover, these immortalized human cells express endogenous TDP1 and TOPO1, allowing for the assessment of the toxic effects of TDP1 mutants with endogenous TDP1 substrate (e.g. TOPO1-DNA) levels in ABSENCE of additionally induced genotoxic stress. It is noteworthy that in our yeast model these conditions do not result in a toxic phenotype [3, 4, 32].

**Figure 3:**
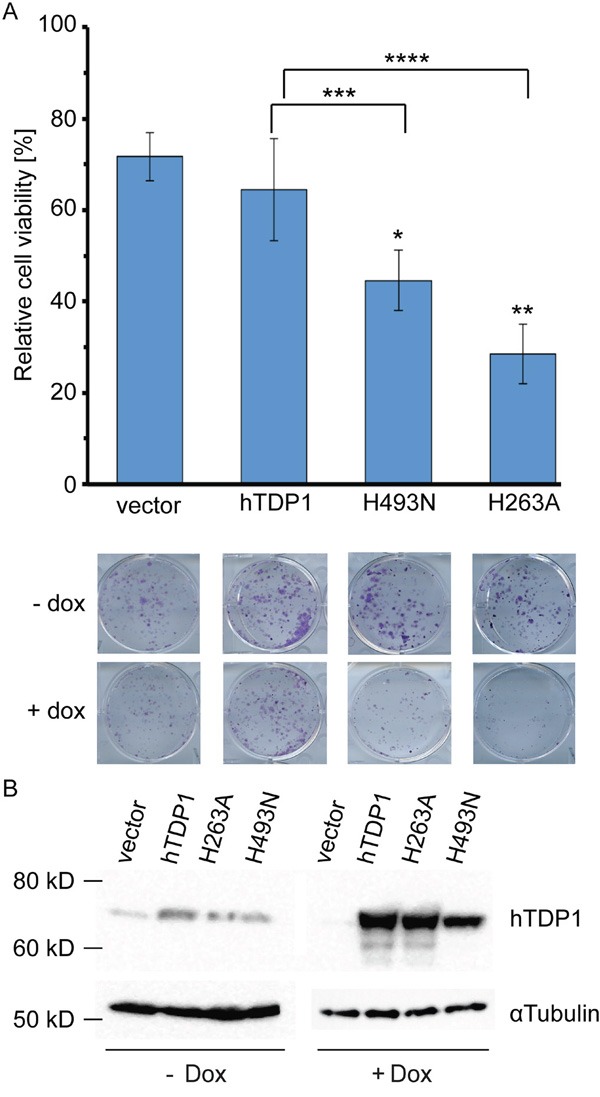
hTDP1H^263^A and hTDP1H^493^N mutants induce toxicity in human cells in absence of genotoxic stress **A**. Stably transfected HEK293 cell lines of hTDP1 with and without Doxycycline (Dox) were incubated at 37°C/5% CO_2_ for 10 days and stained with crystal violet. Colonies were quantified using LI-COR Odyssey Infrared Imager at a fluorescence of 700 nm. Relative cell viability was determined for each cell line induced with Dox relative to its isogenic cells without Dox. Results shown of n=4. A representative photo is shown taken just before quantification of one well of each cell line/condition. Statistics determined by an unpaired student t-test: Vector-hTDP1: no significance. Vector-mutant: *p>0.0007; **p>0.0002. hTDP1-mutant: ***p>0.031; ****(p>0.0087). **B**. Total extracts of 48 hours +/-Dox induction of each stable cell line were resolved on 12% SDS-PAGE and stained with anti-human TDP1. Blot is subsequently stripped and re-stained with anti-αTubulin. For the -Dox 2x more total extract was loaded than for the +Dox total extracts.

To evaluate the cytotoxicity induced by the TDP1 mutants we used an anchorage-dependent colony formation assay. Cell viability of Dox-induced cells expressing the ectopic h*TDP1* allele was determined and plotted relative to the not-induced isogenic cells (no TDP1 expression=100%). Expression of wild-type hTDP1 induced a reduction in cell viability similar to Dox treatment alone (vector control). Conversely, expression of each mutant *TDP1* allele induced a significant reduction in cell viability compared to both vector and hTDP1 expressing cells (Figure 3A). Thus, similar to yTDP1, hTDP1 can be converted into a cellular toxin by mutation of either catalytic histidine.

The levels of ectopic expressed protein are >100-fold over endogenous TDP1 levels, which is similar as reported by Barthelmes et al. [14]. Analysis of hTDP1 protein levels demonstrated a consistent reduction of the hTdp1H^493^N mutant compared to wild type (Figure 3B). This might indicate that the mutant enzyme is trapped on the DNA.

### TDP1 mutants accumulate covalent enzyme-DNA intermediates in cell

**Figure 4:**
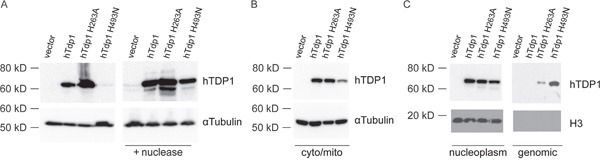
hTDP1 catalytic mutants increase stability of covalent enzyme-genomic DNA intermediates **A**. Band-depletion of stably transfected HEK293 cells. Immunoblot of total cell extracts treated with (+ nuclease) or without micrococcal nuclease. Two times more hTDP1H^493^N total cell extracts were loaded than other samples to emphasize band-depletion. **B-C**. Cell fractionation of the stably transfected HEK293 cells used in (**A**). **B**. Cytosolic/mitochondrial lysate fraction, and **C**. Nuclear fraction split in the nucleoplasm and genomic DNA. All samples were resolved via 12% SDS-PAGE and immunoblotted with anti-hTDP1 and anti-αTubulin (**A-B**); anti-hTDP1 and anti-Histone H3 (**C**).

To determine if the toxicity induced by hTdp1H^493^N and hTdp1H^263^A is due to accumulation of covalent enzyme-DNA complexes, we took a two-pronged approach. *First*, we performed a band-depletion assay in order to observe depletion of the soluble fraction of TDP1 and recovery of the depleted DNA-bound TDP1 fraction via nuclease treatment of the lysate. Immunoblot analysis of cell lysates revealed a significant depletion of hTdp1H^493^N protein levels (even when loaded with twice the amount of cell extract as shown in Figure 4A left panel), suggesting TDP1 is bound to DNA. Indeed, mutant hTDP1 protein levels were recovered upon nuclease treatment (Figure 4A right panel). This is similar to the depletion and recovery of yTdp1H^432^N observed previously [4]. *Second*, we assayed the accumulation of TDP1 protein bound to the genomic DNA by analyzing the cellular distribution of the TDP1 mutants. Equal amounts of cells were harvested and fractionated into the cytosolic/mitochondrial and nuclear fractions. Subsequently, the nuclear fraction was separated into nucleoplasm and genomic DNA to detect TDP1 protein. The TDP1 protein levels in the cytosolic/mitochondrial fraction (Figure 4B) correlated with the band-depletion results (Figure 4A left panel). Analysis of the nucleoplasm and the shredded genomic DNA fractions showed that all ectopic expressed proteins are present in the nucleus, however only the hTdp1H^493^N and hTdp1H^263^A proteins are observed in the genomic DNA fraction (Figure 4C). Anti-histone 3 (H3) blotting showed that the genomic DNA fraction was not contaminated with free, unbound proteins of the nucleoplasm (Figure 4C). This supports that the observed Tdp1-mutant depletion is due to increased levels of covalent Tdp1^mutant^-DNA intermediates.

### Tdp1 catalytic mutants display reduced activity *in vitro*

**Figure 5:**
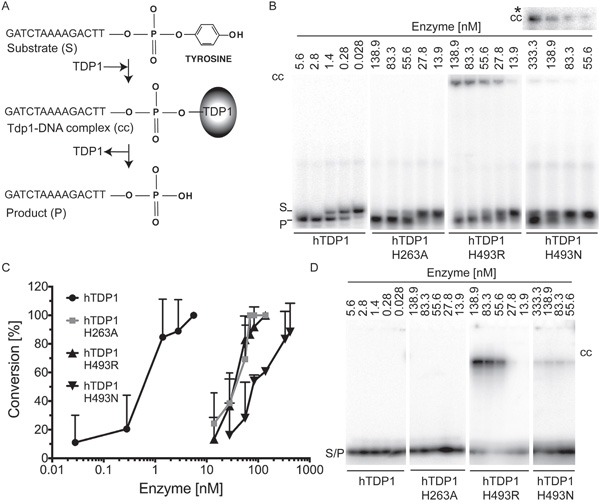
*In vitro* analysis of catalytic activity of hTDP1 enzymes **A**. Schematic of *in vitro* TDP1 catalytic activity assay. 5’-^32^P labeled 14-mer oligonucleotide substrate containing 3’phospho-tyrosine (Substrate (S)) is covalently bound by TDP1 (TDP1-DNA complex (cc)), and subsequently released as 3’phosphoryl end (Product (P)). N-terminally FLAG-tagged hTDP1 protein ranging from 5.6 to 333.3 nM was incubated with 16.7 nM of 5′-^32^P labeled substrate for 10 minutes at 30°C. Reactions were split into two aliquots. **B**. One aliquot was resolved on a denaturing 20% polyacrylamide/8M urea gel, to detect the conversion of the 3′phospho-tyrosine (S) to 3′phosphoryl (P). Stable covalent enzyme-DNA complexes (cc) migrated into gel just under the well. cc* To detect hTDP1H^493^N-DNA cc we increased the exposure time from 1 hour to overnight. **C**. The average and standard deviation of substrate to product conversion (product/[product +substrate]) from (B) were quantitated by ImageStudio Lite (version 3.1.4, LI-COR) of at least three independent experiments. **D**. The other aliquot was resolved by 12% SDS-PAGE to detect covalent enzyme-DNA intermediates (cc). Shown are equal exposure times.

To assess the catalytic activity of the hTDP1 enzymes and possible *in vitro* detection of covalent reaction intermediates, full-length N-terminal FLAG-tagged hTDP1 proteins were expressed in a protease-deficient yeast strain to ensure affinity purified full-length hTDP1 proteins as described in Comeaux et al. [32]. hTDP1 proteins were incubated in reaction mixtures with a ^32^P 5’-end labeled single-stranded oligonucleotide with a 3’phospho-tyrosyl modification as substrate (Figure 5A). Each reaction was split in two samples and analyzed for either *A*) conversion of the 3’phospho-tyrosyl linkage to a 3’phosphoryl product via a denaturing 20%/8 M Urea polyacrylamide gel electrophoresis (PAGE), or *B*) formation of covalent TDP1-DNA reaction intermediates by 12% SDS-PAGE [3]. Relative to wild type hTDP1, where ∼0.45 nM was sufficient to convert 40% of the substrate into a product, hTdp1H^493^N exhibited ∼150-fold lower activity. Surprisingly, hTdp1H^493^R and hTdp1H^263^A exhibited a similar reduction in activity of ∼64-fold less (Figure 5B and 5C). Detection of the hTdp1H^493^N-DNA intermediates in the Urea-PAGE required longer exposure compared to detection the hTdp1H^493^R-DNA intermediates (Figure 5B, *cc represents only the top part of hTdp1H^493^N gel). SDS-PAGE analysis confirmed that the detected protein-DNA intermediates are indeed Tdp1-DNA covalent complexes and showed that hTdp1H^493^N-DNA levels are low compared to hTdp1H^493^R-DNA levels (Figure 5D). However, this is the first time that covalent TDP1-DNA complexes were detected by a Tdp1His^gab^-mutant enzyme (yeast or human) other than the His^gab^Arg substitution. The detected hTdp1H^263^A enzyme activity supports the observed cellular toxicity and the detection of Tdp1H^263^A-DNA covalent intermediates in cells (Figures 3A and 4C, respectively). Moreover, the question remains as to why we detect -reduced- hTdp1H^263^A catalytic activity *in vitro* while it was reported to be catalytically inactive [5, 8].

### hTdp1H^263^A Topo1-dependent toxicity requires His^262^

**Figure 6:**
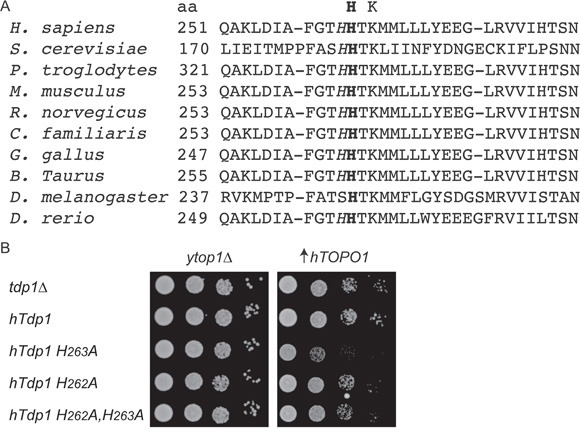
hTDP1H^263^A toxicity involves the conserved adjacent histidine His^262^ **A**. Alignment of a selection of TDP1 orthologs first HxKx_(n)_N motif amino acid (aa) sequences. This motif provides the His^nuc^ (bold) residue; the adjacent His is italicized. UniProt KB/Swiss-Prot protein #: *H sapiens* (Q9NUW8), *S. cerevisiae* (P38319), *P. troglodytes* (G2HG04), *M. musculus* (Q8BJ37), *R. norvegicus* (Q4G056), *C. familiars* (E2REL5), *G. gallus* (F1NSQ5), *B. Taurus* (F1MST1), *D. melanogaster* (Q9VQM4), *D. rerio* (E7FEK0). **B**. *top1*Δ*tdp1*Δ yeast cells co-transformed with vector control (y*top1*Δ) or -h*TOPO1*·U (↑h*TOPO1*) and the indicated YCpGAL1-h*TDP1*·L or its control vector (y*tdp1*Δ). Exponentially growing cells were corrected to an OD_595_ 0.3 and ten-fold serially diluted, spotted onto selective galactose plates, and incubated at 30°C for 4 days.

Despite lacking a nucleophilic histidine, the hTdp1H^263^A enzyme displayed catalytic activity and was able to form a covalent intermediate with genomic DNA to induce cytotoxicity. This led us to postulate the presence of an alternative nucleophilic residue within the catalytic pocket. Sequence analysis of TDP1 orthologs exposed a highly conserved histidine preceding the His^nuc^ as a potential candidate that can act as an alternative nucleophile (Figure 6A). To verify that this histidine, His^262^ in hTDP1, is able to function as an alternative nucleophile, we mutated His^262^ to Ala in wild-type and hTdp1H^263^A mutant alleles. We examined these mutants in our *top1*Δ,*tdp1*Δ yeast strain by co-expression of the h*TDP1* allele with h*TOPO1* as discussed above (Figure 2A). We observed that introduction of H^262^A substitution in hTdp1H^263^A and hTDP1 resulted in cell viability similar to the wild type hTDP1 protein, while hTdp1H^263^A induced cytotoxicity (Figure 6B). This suggests that the catalytic activity of hTdp1H^263^A is mediated by His^262^ functioning as the alternative nucleophile when the His^nuc^ is substituted by Ala, which is not caused by differential protein expression levels (data not shown).

### Topotecan enhances TDP1-induced toxicity

**Figure 7:**
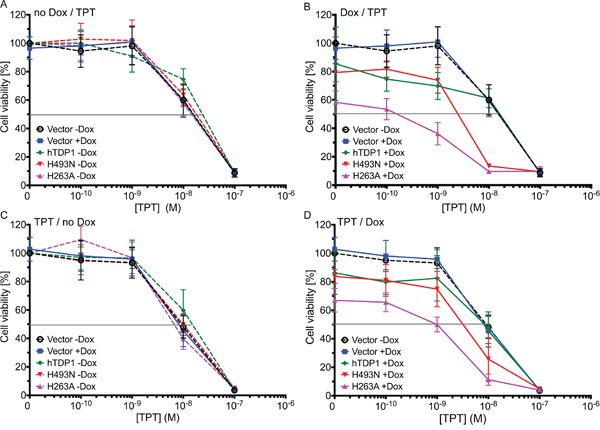
Dysregulated hTDP1 co-treated with topotecan enhances cytotoxicity Exponentially growing stably transfected HEK293 cells with indicated HAh*TDP1* allele or control cells (vector) were seeded 24 hours prior to treatment schedule. **A-B**. Cells were treated with or without Dox 24 hours prior to topotecan (TPT) treatment of 0.1 to 100 nM. **C-D**. Cells were treated with TPT (0.1 to 100 nM) 24 hours prior to ± Dox treatment. 4 days after last treatment cell viability was determined using Allamar blue. Shown is the average and standard deviation of at least 3 independent experiments. **A-C**. no hTDP1 expression was induced; **B-D**. hTDP1 expression was induced by 0.1 μg /mL Dox.

To study the potential of TDP1 as a therapeutic drug target, we tested the schedule dependency of the combinational treatment of Tdp1 mutant expression with topotecan (TPT; reversibly stabilizes Topo1-cc). We tested two treatment schedules. *First*: TDP1 protein expression induced for 4 days, and co-treated with TPT for 3 days. *Second*: TPT treatment for 4 days and TDP1 protein expression induced for 3 days. All stable cell lines showed a similar sensitivity to TPT alone within each schedule (Figure 7A and 7C). Compared to vector control cells, elevated expression of wild-type hTDP1 increased TPT cell sensitivity to sub-nano molar range, while higher TPT concentration induced similar toxicities in both cell lines within each schedule (Figure 7B and 7D; blue and green lines). However, both toxic TDP1 mutant proteins reveal an increased toxicity in combination with TPT over the entire curve in both schedules (Figure 7B and 7D; pink and red lines). TDP1 “poisoning” followed by TPT treatment results in an increase in dynamic range of 4.6- to 35-fold (Table 1). This suggests that the proposed novel therapeutic strategy of molecular targeting the TDP1-cc can result in synergistic toxicity when combined with TPT, but can also be effective by itself (Figure 7).

**Table 1:** IC_50_ of combination treatment of Tdp1 expression and topotecan

Schedule^@^		TDP1-TPT		TPT-TDP1	
Cell line	Dox^$^	IC_50_^#^	Fold Change*	IC_50_^#^	Fold Change*
Vector	-	11.0		9.5	
	+	10.6	1.0	8.8	1.1
hTdp1	-	32.8		13.2	
	+	18.7	1.8	9.4	1.4
H493N	-	11.6		10.0	
	+	2.5	4.6	3.9	2.6
H263A	-	10.6		7.4	
	+	0.3	35.2	1.0	7.4

@ Schedule TDP1-TPT: 4 days with (+Dox) or without (-Dox) TDP1 expression combined with 3 days TPT; TPT-TDP1. Schedule *TPT-TDP1*: 4 days TPT combined with 3 days with (+Dox) or without (-Dox) TDP1 expression; $ -/+ 0.1 μg/ml to induce Tdp1 allele expression; # IC_50_ in nM calculated from the average survival curve (Figure 7) using Prism 7

* Fold change= IC_50_ -Dox/IC_50_ +Dox, indicate fold increase in cell toxicity in combination with indicated TDP1 protein expression and topotecan (TPT).

**Figure 8:**
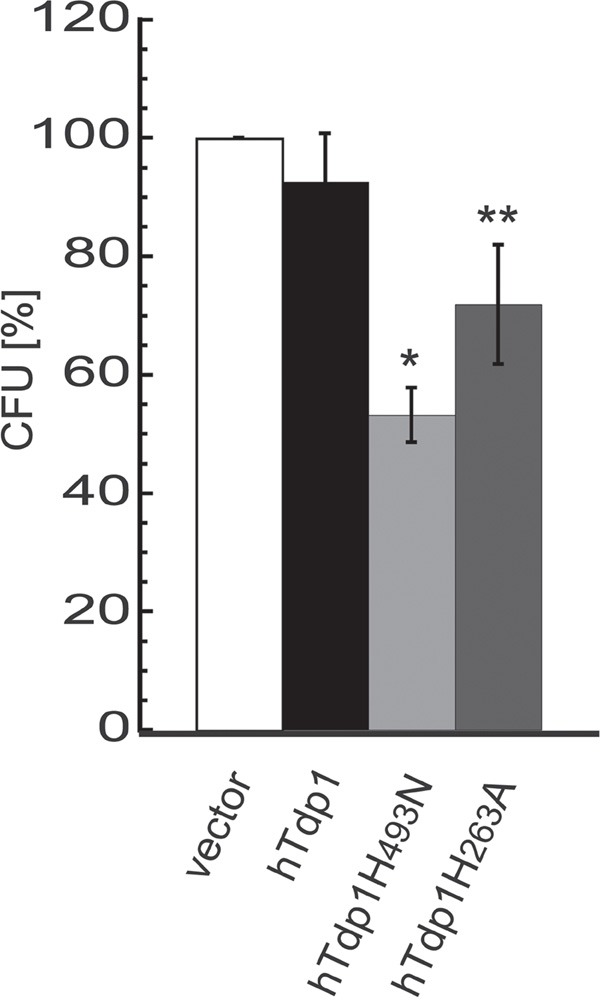
Expression of dysregulated hTDP1 reduces anchorage independent colony formation Stably transfected HEK293 cells with indicated HAh*TDP1* allele were mixed with 0.7% soft-agar medium ± Dox, seeded in 6-well plates and incubated for 21 days. Plates were stained with MTT and colonies were counted. The relative colony forming units (CFU) were determined versus control cell growth (vector). Vector-hTDP1: not significant; Vector-hTdp1^mutant^: *p=0.0001; **p=0.0074, (n=3).

Overall TDP1-induced toxicity increased over time. The combination assays exposed a mild increase in TDP1-induced toxicity (without TPT) between 3-day induction (Figure 7D) and 4-day induction (Figure 7B), while a 10-day incubation period (colony formation assay; Figure 3A) induced even more toxicity. Both these assays are based on anchorage-dependent cell growth, which leads us to the following thought: “Can poisoning of TDP1 via dysregulation of TDP1-activity using either hTdp1H^493^N or hTdp1H^263^A mutants also decrease the anchorage-independent colony formation, which is suggested to be a surrogate for the ability to form cancer metastasis [33, 34]?” Indeed, hTdp1H^263^A significantly reduced colony formation by ∼30% (unpaired student t-test; p=0.0074), while hTdp1H^493^N showed a significant reduction of ∼45% (p=0.0001), compared to vector control (Figure 8). These results suggest that the hTdp1H^493^N mutant is more efficient to prevent anchorage-independent colony formation than the hTdp1H^263^A mutant, which is a reverse outcome compared to the anchorage-dependent colony formation assay (compare Figure 3A with Figure 8). In summary, these results support the potential of targeting TDP1-cc as a novel anti-cancer treatment strategy, which will act synergistically with chemotherapeutics that induce DNA adducts that function as TDP1 substrates.

## DISCUSSION

Our reported yeast observations suggested that TDP1 is a suitable target to turn a DNA repair enzyme into a cellular toxin as a potential novel anti-cancer treatment strategy [3, 4, 32]. TDP1 is unique among DNA repair enzymes due to the requisite formation of a covalent TDP1-DNA reaction intermediate (Figure 1). Other enzymes that remove adducts from the 3’- and 5’-end of DNA nicks do not form a covalent enzyme-DNA complex. For example, TDP2 (also known as TTRAP or EAPII), a multifunctional enzyme that constitutes complementary DNA repair activities with TDP1, hydrolyzing specifically the 5’phospho-tyrosyl linkage. However, TDP2 utilizes a single-Mg^2+^-dependent catalysis, a mechanism that is distinct from TDP1, and lacks the formation of covalent enzyme-DNA intermediates [18, 35–37].

Targeting covalent enzyme-DNA intermediates have been proven to be a very effective anti-cancer strategy. For example, etoposide and TPT specifically stabilize the TOPO2-DNA and TOPO1-DNA covalent intermediates, respectively, to induce cytotoxic DNA lesions [12, 13]. The suitability of the TDP1-DNA complex for molecular targeting to develop novel chemotherapeutics is best illustrated by the identification of an hTdp1His^gab^Arg mutant in patients with the autosomal recessive neurodegenerative disease SCAN1 [28]. This mutant enzyme exhibits a reduced dissociation rate resulting in a more stable hTdp1H^493^R-DNA intermediate and acts as a mild cellular toxin [3, 28, 29]. Surprisingly, this alteration *only* causes a progressive cerebellar atrophy whose symptoms first present during the second decade of life without developing other disorders including immuno-deficiencies, cancers, or myopathy [28, 38]. The mechanistic defect and mild phenotype of this substitution are conserved from human to yeast [3, 4, 28, 29]]. Moreover, we reported that substituting smaller polar or aliphatic side chains such as Asn for yTdp1His^gab^ results in more severe phenotypes that form stable enzyme-DNA reaction intermediates in the cell but not with an artificial *in vitro* oligonucleotide substrate [3, 4]. The observations described above, together with our reported yeast model and the pathobiology of the SCAN1 related TDP1 mutant, support the proposed novel treatment strategy of molecular targeting stabilization of the TDP1-DNA covalent complex for cancer treatment. What's more, most TDP1 substrates are formed by clinically active (chemo-)therapeutics and endogenous reactive oxygen species whose levels are elevated in cancer cells [3, 4, 11, 13, 14, 16–20, 22, 23, 25–27, 39–45].

Indeed, the herein described observations support that molecular targeting to stabilize the TDP1-DNA intermediate is a potential novel cancer treatment strategy. Expression of human Tdp1 His^nuc^Ala and His^gab^Asn mutants in HEK293 cells show growth inhibition and reduced anchorage-dependent and -independent colony formation compared to wild type (Figure 2A, Figure 7B, 7D and Figure 8). Additionally, these hTDP1 mutants show an accumulation of TDP1-genomic DNA reaction intermediates (Figure 4C). These mutants also display a reduced catalytic activity compared to the wild-type enzyme without an easily detectable formation of enzyme-DNA complexes (Figure 5). All these phenotypes are conserved from yeast to human. These results also imply that simple oligonucleotide-based substrates used with *in vitro* reactions are best suited for determining the catalytic activity of TDP1 in contrast to the formation of covalent intermediates, which are best observed in a cellular based assay. This indicates that covalent intermediates are a product of the sum of more physiologically relevant substrates and protein-protein interactions that are not replicated *in vitro*.

Yet our observations surprised us on multiple points. First, we detected catalytic activity *in vitro* and a cellular phenotype with the reported catalytic inactive hTdp1H^263^A mutant [5, 8]. We recently reported a similar observation for the analogous yeast yTdp1H^182^A mutant [32]. We concluded that the His preceding the His^nuc^ is able to rotate into the catalytic pocket in case His^nuc^ is substituted for Ala [3]. Our results suggest that this action is conserved from yeast to human. An hTdp1H^262^AH^263^A double mutant did not reduce cell viability in our yeast model (Figure 6B), which was also observed in an anchorage-independent colony formation assay (data not shown, n=2). Moreover, this suggests that a catalytic inhibitor of TDP1 might not have a significant biological effect in malignancies in which TDP1 is not essential.

For the second point: Although the exact mechanism underlying the toxicity induced by these Tdp1 mutants has yet to be determined (ongoing investigation), it involves the formation of more stable TDP1-DNA reaction intermediates (Figure 4). However, the kinetics of these enzyme-DNA covalent complexes is understudied, which is technically challenging to study in cells. Nevertheless, it is interesting that for the most toxic mutant (hTdp1H^263^A) we detect smaller amount of enzyme-DNA intermediates than for the lesser toxic hTdp1H^493^N mutant (Figure 4C). In general enzyme-DNA intermediates are considered similar in stability, yet the above described data together with our previous observations with yeast Rad9 DNA repair checkpoint deficient strain [32] suggests differently. These catalytic mutants do contain differences as a result of their specific substitutions. Their catalytic pockets are different in electrostatic surface charge and topology (depth, shallowness) as shown for the yeast TDP1 enzymes [3]. This will influence the interaction with the DNA and the conformation of their enzyme-DNA covalent complex, and thus the stability of the complex that correlates with the ability to induce cell lethality.

**Figure 9:**
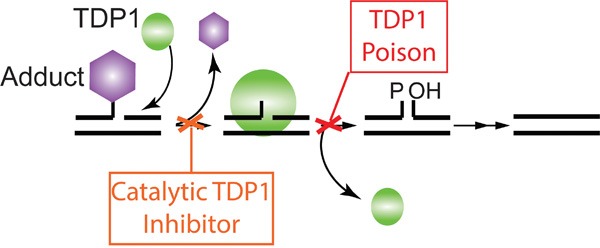
Model of two distinct TDP1 targeted therapeutic approaches Depicted is a schematic representation of TDP1 two-step catalytic cycle. **The first approach**; Catalytic TDP1 inhibitor: This approach prevents repair of chemotherapeutics-induced DNA damage, enhancing their efficacy. **The second approach**; Poisoning or drug-mediated stabilization of the TDP1-DNA covalent complex. This approach represents the ability of molecular stabilization of the obligatory TDP1-DNA reaction intermediate by blocking TDP1 dissociation from the genomic DNA, inducing a more stable DNA-adduct resulting in cell lethality.

Thirdly, the observed phenotype of hTdp1H^263^A and hTdp1H^493^N in the human cell model without additional genotoxic stress was unexpected. Under similar conditions in our yeast model we do not observe toxicity [3, 4]. Moreover, TDP1 activity and as such the level of TDP1-induced toxicity depends on the existences of TDP1 substrates (DNA adducts) since TDP1 cannot function as an endonuclease [3, 27, 45]. In addition, HEK293 cells express endogenous wild type TDP1, which is able to remove TDP1-DNA intermediates. We hypothesize that this heightened sensitivity observed in human cells compared to yeast is likely due to specific differences between the models. Our yeast model is proficient in all DNA repair and DNA damage response pathways, which is not the case for the human cell model. HEK293 cells and other non-cancerous cell lines are immortalized, while cancer cells contain defects that allow them to continuously grow. HEK293 cells are generated by transfection of sheared adenovirus 5 DNA cells, which resulted in p53 inhibition by the large E1B-proteins of adenovirus 5 [46]. Atsumi et al. recently reported that transformed / immortalized cells that harbor mutant or inhibited ARF or p53 are more sensitive to DNA damage induced via replication stress, such as treatment with CPT or hydroxyurea, but not other DNA damage, e.g. doxorubicin or cisplatin [47, 48]. This suggests that the TDP1-induced toxic DNA lesion may result in replication stress to induce the observed cytotoxicity. This suggests that increased stabilization of the covalent TDP1-DNA complex will have a more toxic effect in cells impaired for ARFs/p53 and/or DNA damage response pathway such as cancer cells than in healthy cells. Thus, in contrast to inhibiting TDP1 activity, stabilization of the TDP1-DNA reaction intermediate might be a more efficacious therapeutic approach to treat cancer (Figure 9).

## MATERIALS AND METHODS

### Yeast strains and plasmids

*Saccharomyces cerevisiae* strain KWY4 [*top1Δ,tdp1Δ*] (*MATα, ura3Δ:*:LoxP *his3Δ200 leu2Δ1 trp1Δ63 tdp1Δ*SD *top1Δ::HIS5*) was generated from FY-250 (*MATα, ura3-52, his3A200, leu2Al, trp1Δ63*) by gene replacement of *TOP1* with *HIS5* and the *ura3-52* allele with LoxP-*KAN*^r^-LoxP, followed by CRE-mediated recombination to yield *ura3Δ:*:LoxP, and *TDP1* using *URA3* flanked by gene endogenous *TDP1* 3’UTR repeats, followed by selection on 5-FOA resulting in *tdp1Δ*SD (*Seamless Delete*) to recover *URA3*-selection [3, 49–51]. ECY2 (KWY4, *pep4Δ::TRP1, prb1Δ::URA3*) was generated by gene replacement of *PEP4* with *TRP1* and *PRB1* with *URA3* in KWY4. In all cases, gene deletions were confirmed by PCR and DNA sequencing.

For yeast cell viability studies, h*TDP1* is expressed from the galactose-inducible promoter *GAL1* in YCpGAL1-h*TDP1*·L vectors as described in Gajewski et al. [3]. h*TDP1* mutant alleles were created using the QuikChangeII Site-Directed Mutagenesis kit (Stratagene). h*TOPO1* was expressed from the galactose-inducible *GAL1* promoter of the YCpGAL-h*TOPO1*·U plasmid [52]. For cell viability studies in human cells, the *hTDP1* alleles were N-terminally HA-tagged via PCR and cloned into the pcDNA4/TO expression vector (pcDNA4/TO-HAh*TDP1*) containing a tetracycline /doxycycline-inducible *CMVTetO*_2_ promoter (Invitrogen).

For protein purification from yeast (see also [32]), N-terminal Flag-tagged h*TDP1* constructs, produced by PCR amplification, were subsequently cloned into a YEpGAL4-10GAL1 vector to yield YEpGAL4-10GAL1-FLAGh*TDP1*·L vectors. All *hTDP1* alleles were confirmed by DNA sequencing and PCR primer sequences are available upon request.

### Immunoblotting of yeast cell extracts

As described in He et al. [4], exponentially growing cultures of cells transformed with expressing *TDP1* under the *GAL1* promoter were induced for 6 hours with 2% galactose, corrected to the same OD_595_, and lysed with acid-washed glass beads (Sigma) at 4°C in 50 mM Tris, pH 8.0, 2 mM EDTA, 2 mM EGTA, 10% glycerol, and Complete EDTA-free Protease Inhibitor (Roche). Lysate was boiled in SDS buffer for 10 minutes and aliquots were resolved on a 12% SDS-PAGE, blotted onto PVDF, immunostained with anti-hTDP1 (ab4166, Abcam) and anti-αtubulin (MCA77G, AbD Serotec) antibodies, and visualized by chemiluminescence.

All chemiluminescence (in this manuscript) is detected via film or with the ChemiDoc MP Imaging system and Image Lab 5.2.1 software (Bio-Rad). The film was scanned (CanoScan LiDe 200, Canon), and all images (film scans or ChemiDoc acquired) were processed via Adobe Photoshop CS6 to correct signal levels (if needed) of the complete image before cropping the shown area (band of interest +/- 0.5 to 1 cm margin). Final figures were generated in Illustrator CS6.

### Yeast viability assays

Semi-quantitative colony formation assays were performed as described in He et al. [4]. Briefly, cultures of yeast cells co-transformed with indicated vectors were diluted to OD_595_ of 0.3 in TE buffer (50 mM Tris, 5 mM EDTA) and 10-fold serial diluted. 5 μl aliquots of each dilution were spotted onto selective medium plates containing 2% galactose and incubated for 4 days at 30°C. At least three independent experiments were assayed.

### HEK293T-REx stably transfected cell lines

HEK293T-REx cells (Invitrogen) were transfected with *Xho*1 linearized pcDNA4-CMV-vectors containing the N-terminally HA-tagged h*TDP1* ORF of interest (wild type h*TDP1* or h*TDP1* catalytic mutants) or an empty vector as control, using Lipofectamine 2000 in 6-well plates and incubated overnight at 37°C and 5% CO_2_. Cells were harvested, diluted 1:10, 1:100, 1:1000, and seeded in duplicate in 10 cm dishes for each dilution and incubated at 37°C overnight. 24 hours later selection medium DMEM^zb^ (DMEM supplemented with 10% FBS, 2 mM L-glutamine, 150 μg/ml zeocin (for pcDNA4 vector) and 5 μg/ml blasticidin [T-REx selection]) was added. The selection medium was replaced every 3-4 days without disturbing the cells. Once colonies were formed, colonies were picked using a collection ring and transferred to a 96-well plate. At 90-95% confluence cells were transferred to larger wells until steadily grown in T-75 flasks. Multiple clones were isolated for each introduced h*TDP1* allele and checked for similar HAhTDP1 protein expression. Multiple clones showed similar expression levels, and two arbitrary series of stably transfected clones were selected. One series was used for all assays in triplicate. The other series was used as a control to omit clonal effects due to the integration of the introduced ectopic plasmid. We observed similar outcomes in all tested assays (data not shown). For all used cell lines, the inserted TDP1 cDNAs of the stably transfected lines were verified via sequencing. In addition, all cell lines were authenticated (June 2016) by the UAB Heflin Center Genomic Core Laboratories using the Applied Biosystems AmpFISTR system to screen 15 different small tandem repeat (STR) markers and the Amelogenin locus for gender determination. These STR markers include those genotyped by the ATCC.

### Anchorage-dependent colony formation assays

Exponentially growing stably transfected HEK293 cells with indicated HAh*TDP1* allele or vector control cells were harvested, and 1,000 cells/well in a 6-well plate were seeded in DMEM^zb^. After 24 hours, cells in 2 wells were treated with 0.1 μg/ml doxycycline (Dox) to induce h*TDP1* expression, and cells in 2 other wells were not induced. After incubating for 10 days, cells were fixed with 3.7% paraformaldehyde and stained with 0.025% crystal violet and air-dried. Colonies were quantified using LI-COR Odyssey Infrared Imager at a fluorescence of 700 nm. The readout (light units or LU) of the 2 wells was averaged for each condition and cell line. The relative cell viability was calculated [LU +Dox/ LU -Dox] x100% for each cell line and the average with the standard deviation was determined from at least three independent experiment and plotted. Significance was determined using the unpaired student t-test (Prism 7, GraphPad).

### Immunoblotting of fractionated human cell extracts

Exponentially growing stably transfected HEK293 cells with indicated HAh*TDP1* allele or vector control cells were induced with 1 μg/ml Dox in DMEM^zb^ for 48 hours. Cells were harvested and equal amounts of cells were resuspended in CLB buffer (10 mM HEPES, pH 8.0, 10 mM NaCl, 1 mM KH_2_PO_4_, 5 mM NaHCO_3_, 1 mM CaCl_2_, 0.5 mM MgCl_2_). The lysate was centrifuged at 5000g at 4°C for 5 minutes and cytosolic/mitochondrial fraction (supernatant) was collected. To obtain genomic DNA fraction, the (nuclear) pellet was resuspended in TSE buffer (10 mM Tris, pH 7.5, 300 mM sucrose, 1 mM EDTA), incubated for 30 minutes on ice, and centrifuged at 2300g at 4°C for 5 minutes. The nucleoplasm (supernatant) was collected and the pellet washed twice with TSE buffer and sonicated to shear the genomic DNA to re-solubilize covalently bound proteins. All buffers contain Complete EDTA-free protease inhibitor (Roche). Lysate aliquots were resolved on a 12% SDS-PAGE, blotted onto PVDF and immunostained with anti-hTDP1 and anti-αtubulin or anti-Histone H3 (Ab46765, Abcam) antibodies, and visualized by chemiluminescence. Images are processed as described in “immunoblotting of yeast extracts.”

### Band-depletion of TDP1 protein

Exponentially growing stably transfected HEK293 cells with indicated HAh*TDP1* allele or vector control cells were induced with 1 μg/ml Dox in DMEM^zb^ for 48 hours. Cells were harvested and equal amounts of cells were resuspended in lysis buffer (200 mM NaOH, 5 mM EDTA), incubated for 15 minutes on ice and then neutralized with Neutralization buffer (600 mM Tris, pH 8.0). Half of the cell lysate was added to 6X SDS sample buffer and boiled, while to the other half 6,000 gel units of Micrococcal Nuclease (NEB) was added and incubated at 37°C for 30 minutes. Digestion of genomic DNA was stopped by addition of 6X SDS sample buffer and boiling. Aliquots were resolved on a 12% SDS-PAGE, blotted onto PVDF and immunostained with anti-hTDP1 and anti-αtubulin antibodies, and visualized by chemiluminescence. Images are processed as described in “immunoblotting of yeast extracts.”

### Purification of hTDP1 protein from yeast extracts

N-terminal Flag-tagged h*TDP1* alleles were expressed from YEpGal4-10GAL1-FLAGh*TDP1*·L vectors in protease-deficient *top1Δ,tdp1Δ* cells (ECY2 strain). Exponential cultures were induced with 2% galactose for 6 hours, cells were harvested, and FLAGhTDP1 protein purified as described in [32]. Briefly, cell extracts in HEE/2PI buffer (50 mM HEPES, pH 8.0, 5 mM EDTA, 5mM EGTA, 2PI [2xprotease inhibitor cocktail, Sigma]) were loaded onto SP Sepharose fast flow matrix (GE Life Sciences) and eluted with HEE/2PI/0.4 M NaCl, then affinity purified by anti-FLAG M2 affinity chromatography matrix (Sigma). FLAGhTDP1 was eluted with 3X FLAG peptide in TEEK (50 mM Tris, pH 7.5, 1 mM EDTA, 1 mM EGTA, 100 mM KCl, 1 mM DTT) and concentrated in an Ultracel-30K concentrator (Millipore). Concentration was determined by Bradford-assay (Bio Rad) and purity was determined by sypro-ruby (Bio Rad) staining of hTDP1 fractions resolved by 12% SDS-PAGE.

### hTDP1 *in vitro* activity assay

Activity assays were performed as described in Gajewski et al. [3]. Briefly, 14-mer (5’-GATCTAAAAGACTT-3’) oligonucleotide with a 3’phospho-tyrosine was used as a substrate and an identical oligonucleotide with a 3’-phosphate was used as product control (Midland Certified Reagent Company, Inc.). 5’-^32^P end-labeled oligonucleotides (250 fmol) were incubated in 18 μl reaction buffer (50 mM Tris, pH 7.5, 1 mM EDTA, 100 mM KCl, 2 mM DTT) with indicated amounts of TDP1 for 10 min at 30°C. Reaction samples were split and either combined with USB stop buffer/ 8 M Urea for analysis in 20% PAA/Urea sequencing gel or with 6xSDS-PAGE sample buffer for analysis in 12% SDS-PAGE. Reaction products were visualized by Phosphor-Imager screens, scanned with a Storm 865 scanner (GE Life Sciences), and quantified with Image Studio Software version 3.1.4 LI-COR). All images were processed in Adobe Photoshop CS6 to correct signal levels of the complete image followed by cropping one enzyme's concentration range to obtain separated panels, and final figures were generated in Illustrator CS6.

### Cell viability assay

Exponentially growing stably transfected HEK293 cells with indicated HAh*TDP1* allele or vector control cells were seeded at 2000 cells/well in 96-well plates. The following day cells were treated with or without 0.1 μg/ml Dox in DMEM^zb^ and 24 hours later with various concentrations ranging from 1-100 ng/ml of topotecan (TPT) or vice versa depending on the treatment schedule (+/- Dox [to induce hTDP1 expression] followed by TPT or TPT followed by +/-Dox). After 4 days of incubation, cells were treated with 10% (v/v) AlamarBlue (Invitrogen) and incubated at 37°C for 2 hours. Plates were read on the SpectraMax M5 plate reader (Molecular Devices) at a fluorescence of 530/590 nm. Relative cell viability was determined relative to DMSO (vertical) treatment. Four wells were used per condition in each experiment and the average and standard deviation of at least three independent experiments were plotted and analyzed using Prism 7 (GraphPad).

### Anchorage-independent-colony formation assays

Exponentially growing stably transfected HEK293 cells with indicated HAh*TDP1* allele or vector control cells were harvested, and 30,000 cells were mixed with DMEM^zb^ /0.7% agarose, with or without 1 μg/ml Dox, and plated onto DMEM^zb^ /2% agarose with or without 1 μg/ml Dox in 6-well plates and incubated for 21 days, then stained with MTT (5 mg/ml in PBS) overnight and colonies counted. Each cell line and condition (+/- Dox) was seeded in duplicate. The relative colony forming units (CFU) were determined versus vector control for each condition (with or without Dox induction). Plotted is the average and standard deviation of three independent experiments for each condition. The no Dox results showed no significant difference versus vector control (data not shown). Significance was determined using the unpaired student t-test.
